# Sensorineural Hearing Loss following Carbon Monoxide Poisoning

**DOI:** 10.1155/2012/231230

**Published:** 2012-05-07

**Authors:** Joseph P. Pillion

**Affiliations:** ^1^Department of Audiology, Kennedy Krieger Institute, Baltimore, MD 21205, USA; ^2^Department of Physical Medicine and Rehabilitation, Johns Hopkins University School of Medicine, Baltimore, MD 21205, USA

## Abstract

A case study is presented of a 17-year-old male who sustained an anoxic brain injury and sensorineural hearing loss secondary to carbon monoxide poisoning. Audiological data is presented showing a slightly asymmetrical hearing loss of sensorineural origin and mild-to-severe degree for both ears. Word recognition performance was fair to poor bilaterally for speech presented at normal conversational levels in quiet. Management considerations of the hearing loss are discussed.

## 1. Introduction

Sensorineural hearing loss has been reported with both acute [1–4] and chronic [3, 5, 6] carbon monoxide poisoning.Other neurological sequelae such as visual failure, object agnosia, finger agnosia, temporospatial disorientation, dysphasia, Parkinsonism, epilepsy, incontinence, and mental changes ranging from cognitive delays to psychosis have been reported [7]. Other investigators have noted the presence of peripheral neuropathy [8]. Hearing loss ranging from slight to severe [6] has been reported. In some cases partial or complete recovery from the hearing loss has been reported [3, 5]. Hearing loss in CO poisoning is typically bilateral, although unilateral hearing loss has been reported [9]. The present case study is of a 17 year old with acute exposure to carbon monoxide who developed a moderate-to-severe sensorineural hearing loss as well as other neurological impairments.

## 2. Case Presentation

The patient was a 17-year-old male who was in previously good health with no history of hearing loss or significant medical issues. He was found at home and was unresponsive due to carbon monoxide (CO) poisoning. He was sleeping on the family couch in the living room on the first floor. Due to a hurricane-related power failure, a diesel generator was running in the family's connected garage. The patient was later found unconscious on the couch. Other family members were also found unconscious on the scene; not all of the family members survived the CO poisoning. The patient had been unconscious for an unknown period of time and was taken to a shock trauma center by emergency personnel arriving at the scene. He was intubated due to his level of consciousness.

 Upon arrival at the shock trauma center, a CT scan of the head showed no signs of obvious pathology. A chest X-ray showed evidence of a small focal consolidation on the lower right lobe. There was evidence of mild bilateral calcification suggesting pulmonary edema. On the evening of his admission, he was taken to a hyperbaric oxygen chamber. The day after his admission an MRI was administered which showed some evidence of anoxic brain injury. He was found to have diffuse white matter cytotoxic edema which involved the bilateral frontal, parietal, and temporal lobes. He was later extubated while at the shock trauma center. By the time of his discharge from the shock trauma center he was able to follow some simple commands and was able to pass a PO diet. However, he remained nonverbal. He was discharged when believed to be stable after nine days to an inpatient brain injury rehabilitation facility. Prior to his transfer his auditory status was not formally assessed.

 Upon admission to the rehabilitation facility he was arousable for short periods of time and was able to respond to yes-no questions with a head nod. Initially he was uncooperative with physical examination. He would attempt to verbalize and was able to follow one-step commands. Overall he had generalized weakness and was flaccid on the left with an increase in tone on the right. His sensation was intact on all four extremities as were deep tendon reflexes. He was diagnosed at admission with gait dysfunction, dysphasia, and generalized weakness. He received intensive physical, occupational, and speech language pathology therapies during his admission where he remained for 23 days. Initially, he appeared to be aphasic. Over the course of his admission his alertness level normalized, and he became more oriented. His speech and language skills reemerged, and he was able to tolerate a regular diet. He was discharged to home after 16 days with 24-hour supervision required. His hearing was never formally assessed during the admission.

He subsequently received 13 days of intensive physical therapy, occupational therapy, and speech and language therapy at a day program three days a week. He also received an in-depth neuropsychological evaluation prior to returning to school as well as grief counseling. He was diagnosed at this time with mild mixed aphasia and mild-moderate cognitive deficits. While receiving speech and language pathology services at the day rehabilitation facility, the patient complained of difficulty in hearing to the speech and language pathologist and was referred for an audiological evaluation.

**Figure 1:**
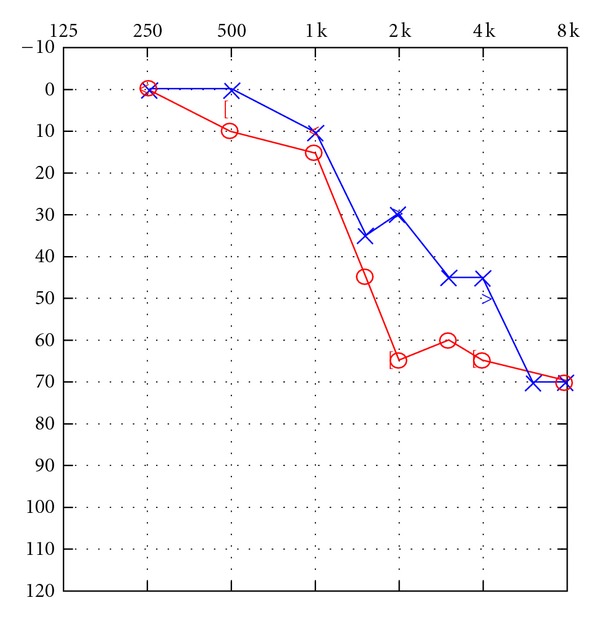
Initial audiogram obtained 54 days after CO poisoning.

**Figure 2:**
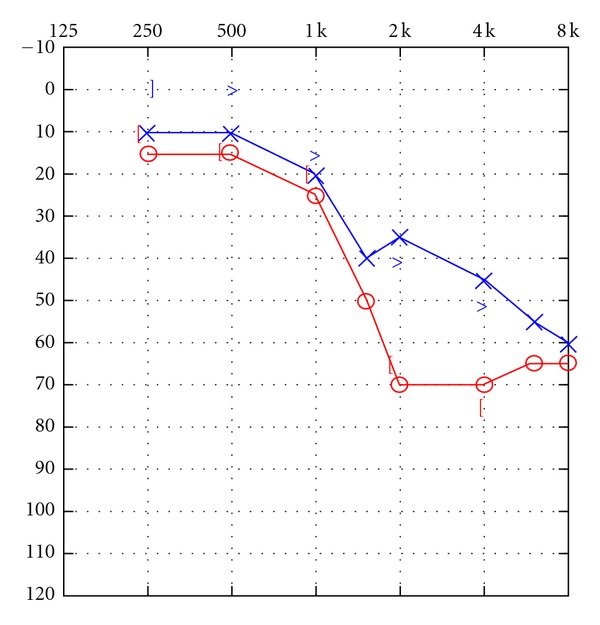
Follow-up audiogram obtained one month subsequent to initial audiogram.

The patient was seen for an audiological evaluation 54 days after his exposure to CO. He denied tinnitus. The obtained audiogram is shown in Figure 1. Findings indicated the presence of a bilateral hearing loss of sensorineural origin. For the right ear, findings indicated the presence of normal sensitivity from 250 to 1000 Hz, sloping to a moderate hearing loss at 1500 Hz and a moderate-to-severe hearing loss at 2000 Hz and above. For the left ear, findings indicated the presence of normal sensitivity at 250–1000 Hz, sloping to a mild hearing loss at 1500–2000 Hz and a moderate-to-severe hearing loss at 3000 Hz and above. Bone conduction thresholds interweaved with air conduction thresholds indicating the impairment to be sensorineural in origin bilaterally. Speech reception thresholds were in good agreement with frequency-specific findings bilaterally. Word recognition performance in quiet was fair-poor bilaterally at a level 40 dB above the speech reception threshold but improved to fair-good at 80 dB SL. There was no PB rollover for either ear. Word recognition performance was not disproportionately impaired relative to the degree of pure-tone sensitivity loss [10]. Tympanometry revealed normal tympanic membrane/middle ear system mobility bilaterally. Acoustic reflexes were absent or present at elevated sensation levels for ipsilateral stimulation of both ears. Measurements of transient evoked otoacoustic emissions were undertaken for stimulation of both ears. TEOAEs were absent for both ears from 1000 to 4000 Hz, indicating the presence of outer hair cell dysfunction bilaterally. Subsequent audiological assessment one month later revealed no significant changes in the patient's pure tone sensitivity as shown in Figure 2. Hearing aids were recommended, and the patient was provided with an FM system during the duration of his rehabilitation activities at the day program rehabilitation facility. Staff interacting with the patient in his therapies noted some immediate improvement in his performance. He was subsequently issued binaural hearing aids which were to be coupled with an FM system in his school setting.

## 3. Discussion

The present patient demonstrated a bilateral mild-to-severe sensorineural hearing loss in the high-frequency region secondary to CO poisoning. The absence of otoacoustic emissions indicates that the impairment in co poisoning is present at the level of the outer hair cells and is not confined to neural levels as has been suggested previously [11]. Absent otoacoustic emissions have also been reported in one other case of CO poisoning [5]. Word recognition performance was fair to poor at normal conversational levels but was not considered disproportionately impaired relative to pure tone sensitivity. The patient was assessed at 2 and 3 months after CO exposure, and no recovery of auditory sensitivity was detected at that point in his overall recovery. Nearly complete [1, 5] or partial recovery [3] of hearing loss has been reported in some cases in the literature. Tinnitus has been reported in hearing loss secondary to CO poisoning [8] but was not reported in our patient. While dizziness as well as vestibular impairment has been documented in electronystagmography examination in CO poisoning [12], the patient in this report denied experiencing any dizziness symptoms. A notched shaped audiometric configuration has been reported as a characteristic of hearing loss in acute CO poisoning [4] and has been noted in some case reports [2, 3] but was not seen in the present case or in others reported in the literature [1, 8]. The patient's hearing loss was bilateral as has been typically reported in the literature with only one exception [9].

 The mechanism responsible for hearing loss in CO poisoning has not been definitively established but is likely due to hypoxic effects on tissue [5]. The damage may be due to the cochlea, auditory nerve, and central auditory pathways [5]. Hypoxia results from the conversion of oxyhemoglobin to carboxyhemoglobin. More central effects to the globus pallidus have been reported in acute CO poisoning [1].

Audiological assessment is not routinely offered in cases with brain injury at some rehabilitative centers. This case illustrates the importance of audiological assessment in cases of brain injury secondary to carbon monoxide poisoning. This patient was provided with intensive rehabilitative services without consideration of the presence of the possibility of a communicatively handicapping hearing loss. When he was provided with some assistance to address the hearing loss, some immediate improvement was noted in his ability to respond appropriately to communication.
